# Surgical treatment of transcatheter aortic valve infective endocarditis

**DOI:** 10.1007/s12471-020-01494-y

**Published:** 2020-10-06

**Authors:** P. G. Malvindi, S. Luthra, S. Sarvananthan, A. Zingale, C. Olevano, S. Ohri

**Affiliations:** Wessex Cardiothoracic Centre, University Hospital Southampton, Southampton, UK

**Keywords:** TAVI, endocarditis, aortic valve, reoperation, aortic valve replacement

## Abstract

There is growing interest in infections occurring after transcatheter aortic valve implantation (TAVI). The incidence, and clinical and anatomical features suggest many similarities with prosthetic valve endocarditis. The survival of patients with an infected TAVI prosthesis is generally poor; however, only a minority of them (10%) have undergone treatment with surgical explantation of the infected prosthesis. A literature search was performed using online databases. Papers reporting surgical treatment of TAVI prosthesis infections were retrieved, focusing on pre- and intraoperative characteristics and early outcome. Thirty-seven papers ultimately provided information on 107 patients. Their mean ± standard deviation (SD) age was 76 ± 8 years and 72% were male. The mean ± SD time interval between the TAVI procedure and reoperation was 10 ± 10 months. Annular abscess formation was described in 34% of cases and mitral valve involvement in 31%. All patients underwent TAVI prosthesis explantation and surgical aortic valve replacement; concomitant mitral valve replacement was necessary in 22% of cases. Postoperative in-hospital mortality was 28%. Surgical explantation of infected TAVI prostheses was associated with a high postoperative mortality, although these initial experiences included elderly and high-risk patients. Considering the expansion of TAVI procedures towards younger and lower-risk patients, surgical treatment of TAVI endocarditis may represent the best option for a life-saving procedure.

**Electronic supplementary material:**

The online version of this article (10.1007/s12471-020-01494-y) contains supplementary material, which is available to authorized users.

## Introduction

Infective endocarditis is still associated with high mortality and morbidity rates [1, 2]. Contemporary population surveys reported an annual incidence of 3–7 per 100,000 person-years [3–5]. Prosthetic valve endocarditis is associated with a much higher risk of 0.3–1.2% per patient-year [6–10]. Substantial heterogeneity exists between studies due to differences in population characteristics, risk profile, predisposing factors, microbiological diagnosis and different definitions of infective endocarditis [11–15]. Studies with a longer follow-up time in which large cohorts of patients were analysed, showed that infective endocarditis is not uncommon and reported a cumulative incidence of TAVI prosthesis infection of 5% during the first 5 years [12, 16, 17]. Several risk factors have been associated with TAVI endocarditis [13–15, 18–20], and alternative imaging tools have been proposed for a prompt and correct diagnosis [11, 14, 21]. Robust evidence for effective treatment strategies that improve the prognosis is still lacking despite a better understanding of the incidence, causes and means of prevention [22].

Most patients with TAVI endocarditis (90%) receive conservative management, resulting in a dismal early outcome with high in-hospital mortality and poor short-term survival [11, 13, 14]. Surgical treatment with explantation of the TAVI prosthesis has not shown superior survival. Experience with treatment remains limited, and most patients are deemed inoperable or at excessive surgical risk in an elective setting [13, 18, 23]. Expanding indications to include intermediate- to low-risk patients for TAVI mandate a better understanding of the treatment of TAVI endocarditis, comparison of medical versus surgical options and well-defined treatment pathways.

The aim of our study was to review the patient characteristics, microbiology, underlying causes and outcomes of patients who underwent explantation of TAVI valves due to infective endocarditis.

## Methods

### Literature search

A literature search was performed using online databases and web-based search engines (PubMed, Google Scholar, Google, ResearchGate) to obtain research articles about infection of TAVI prostheses. The following keywords were used: ‘TAVI’, ‘transcatheter aortic valve’, ‘TAVR’, ‘infection’, ‘endocarditis’, ‘explant’, ‘explantation’ and ‘retrieval’.

Two independent reviewers (SS and AZ) identified and assessed the studies for eligibility. We applied the following inclusion criteria: (1) study in adult human subjects who underwent TAVI prosthesis explantation due to infective endocarditis; (2) paper reporting results of TAVI prosthesis explantation due to infective endocarditis; (3) paper written in English; (4) no restriction regarding the date of publication (last search performed in June 2020); and (5) no restriction regarding type of prosthesis. Exclusion criteria were: (1) study in adult human subjects and paper reporting results of TAVI endocarditis treated conservatively; (2) study in adult human subjects and paper reporting results of TAVI endocarditis treated with interventional procedure; and (3) paper not reporting details of patient characteristics or outcomes. All differences of opinion and divergences were resolved by consensus after discussion with a third reviewer (PGM).

Eligible full-text papers were cross-referenced, and patient characteristics of interest and relevant outcomes were extracted from the included studies. Extracted data included the following:

- Number of patients, type of prosthesis, and interval time between TAVI implantation and explantation;
- Patient characteristics: age, gender, previous cardiac surgery, clinical presentation, result of cultures, risk score, presence of vegetation(s), presence of annular abscess, involvement of mitral valve, associated complications and type of surgical operation performed;
- Outcomes: in-hospital mortality, details of postoperative course and survival.

**Fig. 1:**
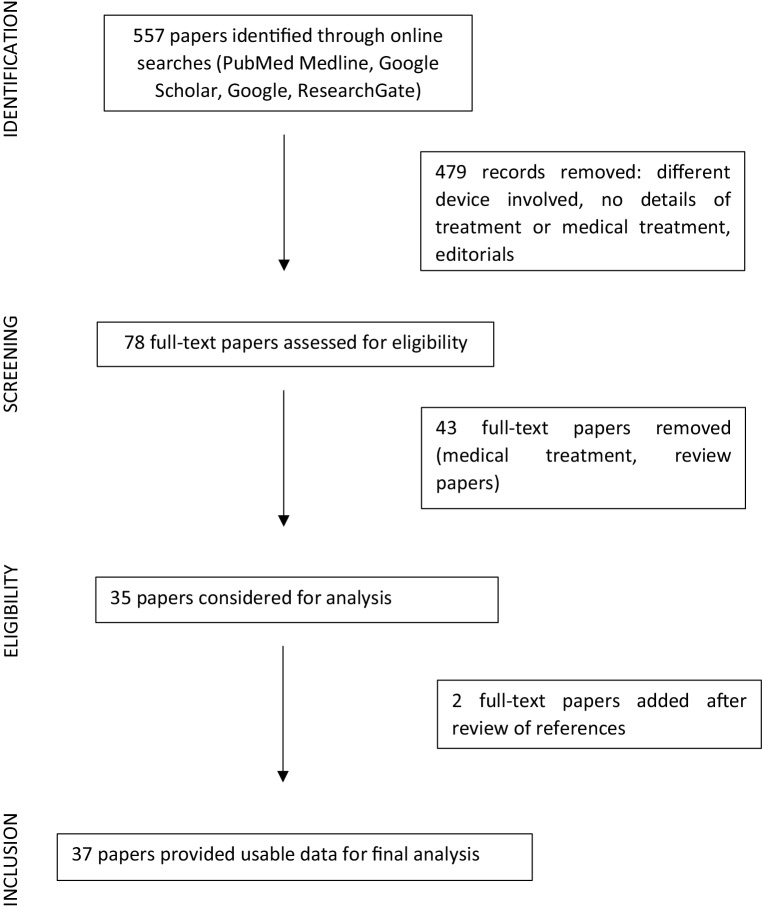
Flowchart of literature search strategy, according to rules specified by Preferred Reporting Items for Systematic Reviews and Meta-Analyses (PRISMA)

Patient characteristics are presented as mean ± standard deviation, median (range), or number and percentage. Fig. 1 represents a search flowchart that was created according to the rules specified by the Preferred Reporting Items for Systematic Reviews and Meta-Analyses (PRISMA) [24]. The PRISMA checklist, including the PubMed search, can be found in the Electronic Supplementary Material.

## Results

### Study selection

A total of 557 papers were screened and, after removal of 479 records, 78 full-text papers were assessed for eligibility. We cross-referenced reports to include any missed studies. Finally, 37 papers (7 series and 30 case reports) provided data on 107 patients, who were included in the study (Fig. 1). A summary of the studies is included in Tab. 1 in the Electronic Supplementary Material.

### Preoperative characteristics

The mean age was 76 ± 8 years. Most patients were male (72%). A previous cardiac operation was performed in 15% of the cases. For 64 patients, the interval time between the TAVI procedure and the reoperation was provided: the mean time was 10 ± 10 months (median 8 months; range 1–52).

Clinical presentation was characterised by persistent fever or sepsis in 74% of cases and by refractory heart failure in 26%. Embolic complications were described in 35% of cases. Echocardiographic evidence of infection was most commonly seen as vegetations (65%), annular abscess (34%) and additional mitral valve involvement (31%). Tab. 1 in the Electronic Supplementary Material details the preoperative, operative and outcome data available for all full-text papers included in the final analysis. The most common organisms were coagulase-positive staphylococci, streptococci and enterococci; no growth was observed in 5% of cases. Details regarding microbiological findings are reported in Tab. 1.

**Table 1:** Microbiological findings^a^

Microorganism	Frequency (%)
*Staphylococcus* spp	33
– Coagulase-positive/*S. aureus*	18
– Coagulase-negative	16
*Streptococcus* spp	24
*Enterococcus* spp	23
Fungi	7
*Corynebacterium* spp	3
Other Gram-positive bacteria	2
Other Gram-negative bacteria	2
Negative	5

^a^Data available for 61/107 patients

### Surgical treatment and early outcome

All patients underwent TAVI prosthesis explantation and surgical aortic valve replacement; Tab. 2 shows details of the type of TAVI prosthesis. Associate procedures were annular patch reconstruction in 11% of cases, aortic root replacement in 13% and mitral valve replacement in 22% (Tab. 1 in the Electronic Supplementary Material).

**Table 2:** Details of TAVI prostheses^a^

Type of valve	Frequency (%)
Balloon-expandable	68
– Sapien	30
– Sapien XT	15
– Sapien 3	12
– Lotus	1
– Not defined	8
Self-expandable	32
– CoreValve	15
– Jena Valve	2
– Symetis	2
– Portico	1
– Not defined	12

*TAVI* transcatheter aortic valve implantation

^a^Data available for 59/107 patients

Overall postoperative in-hospital mortality was 28%; this percentage was 22% for patients who had not undergone a previous cardiac operation and 33% for patients who needed a redo cardiac procedure. Follow-up data were provided in 12 papers; in the series of 20 patients by Mangner et al. [23], the mortality rate through the first year after surgery was 15%. Eleven case reports reported survival data, with 10 patients being alive at a median time of 12 months (range 6–45).

## Discussion

Infection of TAVI prostheses is not uncommon, with a reported incidence of 0.5–1.6% per year in populations followed up to 5 years; the concomitant survival rate is 40–50% [12, 54–56]. Review papers [18, 57, 58] and analyses of national and international registries [11–14, 16] have started delineating the common features of TAVI prosthesis infection and have ultimately highlighted two important findings: patients with TAVI endocarditis suffer a dismal early outcome with a mortality of 40–70% at 1 year after the diagnosis, and surgical explantation of the infected prosthesis was performed in only 2–14% of cases despite clear indications for surgical intervention in more than 80% of patients [11, 13, 14, 16, 41, 59].

Reasons for not operating include a remarkably high clinical or surgical risk, need for additional procedures, advanced age of the patient and limited life expectancy, multiorgan failure, septicaemia and dismal long-term prognosis. Within the limitations of including small series and case reports, we found an early survival rate of more than 70% after surgical retrieval of infected TAVI prostheses. Early postoperative mortality is strictly associated with the risk profile of these patients (active infection, age, 15% needing a redo procedure) and, as per initial TAVI practice, with the coexistence of unfavourable clinical or anatomical features for conventional surgical aortic valve replacement, making these patients already at a high risk for isolated aortic valve disease in an elective setting. Local complications are also common in TAVI endocarditis, as we found evidence of periannular abscess formation and mitral involvement in more than one third of the patients. These findings confirm previous observational studies reporting periannular abscess and mitral valve involvement in about 12–25% and 22–25% of patients with TAVI endocarditis, respectively [11, 58]. A similar rate of periannular complications has been reported for surgical aortic prosthesis infective endocarditis, with 50–60% of patients presenting with an annular abscess, a fistula or a false aneurysm [60]. These cases represent a medical and technical challenge, as they are associated with a more severe or advanced infective process and require a complex surgical repair; we showed that a full root replacement was performed in 13% of cases and mitral valve surgery was required in 22% of patients.

The microorganisms involved in the infective process were the common pathogens found in elderly populations. Both staphylococci and streptococci were isolated in about 30% of cases and *Enterococcus* was found in one-fourth of the patients. These results fully align with previous findings in patients with TAVI infective endocarditis [11, 16, 58, 59].

Follow-up data were provided in 12 out of the 37 papers included in our analysis. The only series reporting the 1‑year survival showed 15% of post-discharge mortality during the first year [23]. Two previous studies compared the survival of patients with TAVI endocarditis who received medical therapy vs surgical operation [18, 23]. Those who underwent medical treatment were older, had a higher Society of Thoracic Surgeons score and more often had severe chronic kidney disease. On the other side, patients who were treated with TAVI prosthesis retrieval and surgical aortic valve replacement more frequently presented with significant aortic regurgitation, annular abscess and mitral valve involvement. No statistical differences were found in terms of mortality and survival between the two treatment options, a result which was confirmed after comparison of matched populations [23]. However, the follow-up time was limited to 3 months [18], and 12 months [23]. Patients operated for surgical prosthetic valve infective endocarditis usually sustain a non-negligible rate of mortality in the early postoperative period, but during the first year, a drop in survival curves is usually seen due to infection relapse or lack of recovery. A longer follow-up period may show a larger difference in survival curves in favour of surgical explantation of the TAVI prosthesis.

### Study limitation

The main limitation of this review is the inclusion of small series and case reports, which could have biased this initial picture of surgical treatment for TAVI prosthesis infection. Long-term survival data were lacking in most case series. Collation and assimilation of data on individual outcomes and a formal meta-analysis could not be carried out, making it difficult to draw meaningful conclusions regarding the effectiveness of the surgical approach. However, we do not believe that our results are far different from reality. They seem to be in line with the more robust findings coming from surgery for infected aortic valve prostheses, for which, due to technical and medical challenges, we could expect an in-hospital mortality rate up to 15–20% [64–66]. Furthermore, a recent preliminary analysis of the Society of Thoracic Surgeons database reported a similar postoperative in-hospital mortality (29.3%) in patients who underwent TAVI prosthesis retrieval and surgical aortic valve replacement for infective endocarditis (oral presentation at 2020 ACC World Congress of Cardiology [67]).

The main reason for denying a surgical procedure in almost 90% of patients with a TAVI prosthesis infection is probably the previous decision to avoid a conventional operation in favour of a transcatheter procedure and the coexistence of important clinical comorbidities and/or anatomical difficulties. Patients’ age could have played an important role as well, but the low survival rate registered after medical therapy suggests maximising the effort in providing a surgical solution. The majority of patients requiring TAVI prosthesis explantation would also not need a redo sternotomy; thus, they would have a lower risk of re-entry complications and bleeding than patients with a previously implanted surgical prosthesis. Local aggressiveness of the infective process results in periannular abscess formation and, more importantly, in the involvement of the anterior mitral valve leaflet and the occurrence of new mitral regurgitation. This last issue is a peculiar finding in TAVI endocarditis and, rather than being considered a further operative risk factor, should result in a prompt indication for a surgical operation.

## Conclusion

The surgical treatment of TAVI infective endocarditis is associated with a high in-hospital mortality rate. However, these initial experiences included elderly and high-risk patients presenting with local and systemic infective complications and often requiring a complex surgical repair. Despite the medical and technical challenges associated with this disease, a radical and prompt surgical treatment with the resolution of sepsis and heart failure can provide acceptable mid-term outcomes and could be the best option for a life-saving procedure.

## Caption Electronic Supplementary Material

PRISMA 2009 Checklist

PubMed research June 2020

Tab. 1 List of full-text papers included in the review

## References

[1] Thuny F, Grisoli D, Collart F, Habib G, Raoult D (2012). Management of infective endocarditis: challenges and perspectives. Lancet.

[2] Habib G, Lancellotti P, Antunes MJ (2015). 2015 ESC Guidelines for the management of infective endocarditis: The Task Force for the Management of Infective Endocarditis of the European Society of Cardiology (ESC) Endorsed by: European Association for Cardio-Thoracic Surgery (EACTS), the European Association of Nuclear Medicine (EANM). Eur Heart J.

[3] Duval X, Delahaye F, Alla F (2012). AEPEI Study Group. Temporal trends in infective endocarditis in the context of prophylaxis guideline modifications: three successive population-based surveys. J Am Coll Cardiol.

[4] Federspiel JJ, Stearns SC, Peppercorn AF, Chu VH, Fowler VG (2012). Increasing US rates of endocarditis with Staphylococcus aureus: 1999–2008. Arch Intern Med.

[5] Selton-Suty C, Célard M, Le Moing V (2012). AEPEI Study Group. Preeminence of Staphylococcus aureus in infective endocarditis: a 1-year population-based survey. Clin Infect Dis.

[6] Thornhill MH, Jones S, Prendergast B (2018). Quantifying infective endocarditis risk in patients with predisposing cardiac conditions. Eur Heart J.

[7] Agnihotri AK, McGiffin DC, Galbraith AJ, O’Brien MF (1995). The prevalence of infective endocarditis after aortic valve replacement. J Thorac Cardiovasc Surg.

[8] Wang A, Athan E, Pappas PA (2007). International Collaboration on Endocarditis-Prospective Cohort Study Investigators. Contemporary clinical profile and outcome of prosthetic valve endocarditis. JAMA.

[9] Bashore TM, Cabell C, Fowler V (2006). Update on infective endocarditis. Curr Probl Cardiol.

[10] Moreillon P, Que YA (2004). Infective endocarditis. Lancet.

[11] Bjursten H, Rasmussen M, Nozohoor S (2019). Infective endocarditis after transcatheter aortic valve implantation: a nationwide study. Eur Heart J.

[12] Butt JH, Ihlemann N, De Backer O (2019). Long-term risk of infective endocarditis after transcatheter aortic valve replacement. J Am Coll Cardiol.

[13] Regueiro A, Linke A, Latib A (2016). Association between transcatheter aortic valve replacement and subsequent infective endocarditis and in-hospital death. JAMA.

[14] Amat-Santos IJ, Messika-Zeitoun D, Eltchaninoff H (2015). Infective endocarditis after transcatheter aortic valve implantation: results from a large multicenter registry. Circulation.

[15] Olsen NT, De Backer O, Thyregod HG (2015). Prosthetic valve endocarditis after transcatheter aortic valve implantation. Circ Cardiovasc Interv.

[16] Kolte D, Goldsweig A, Kennedy KF (2018). Comparison of incidence, predictors, and outcomes of early infective endocarditis after transcatheter aortic valve implantation versus surgical aortic valve replacement in the United States. Am J Cardiol.

[17] Søndergaard L, Ihlemann N, Capodanno D (2019). Durability of transcatheter and surgical bioprosthetic aortic valves in patients at lower surgical risk. J Am Coll Cardiol.

[18] Pericas JM, Llopis J, Cervera C (2015). Infective endocarditis in patients with an implanted transcatheter aortic valve: clinical characteristics and outcome of a new entity. J Infect.

[19] Mangner N, Woitek F, Haussig S (2016). Incidence, predictors, and outcome of patients developing infective endocarditis following transfemoral transcatheter aortic valve replacement. J Am Coll Cardiol.

[20] Martinez-Selles M, Bouza E, Diez-Villanueva P (2016). Incidence and clinical impact of infective endocarditis after transcatheter aortic valve implantation. EuroIntervention.

[21] Salaun E, Sportouch L, Barral P-A (2018). Diagnosis of infective endocarditis after TAVR: value of a multimodality imaging approach. JACC Cardiovasc Imaging.

[22] Monteagudo Ruiz JM, Zamorano Gómez JL (2019). Endocarditis after transcatheter aortic valve implantation: a new fiend we hardly know. Eur Heart J.

[23] Mangner N, Leontyev S, Woitek FJ (2018). Cardiac surgery compared with antibiotics only in patients developing infective endocarditis after transcatheter aortic valve replacement. J Am Heart Assoc.

[24] Tricco AC, Lillie E, Zarin W (2018). PRISMA extension for scoping reviews (PRISMA-scR): checklist and explanation. Ann Intern Med.

[25] Santarpino G, Fischlein T, Pfeiffer S (2013). Prosthetic valve endocarditis 6 months after transcatheter aortic valve implantation. G Ital Cardiol.

[26] Seok Koh Y, Hyoung MM, Jo HK, Wook KH (2014). Infective endocarditis in transcatheter aortic valve implantation. Eur J Cardiothorac Surg.

[27] Wilbring M, Tugtekin SM, Matschke K, Kappert U (2014). Surgery for fulminant prosthetic valve endocarditis after transapical transcatheter aortic valve-in-valve implantation. Thorac Cardiovasc Surg.

[28] Ahmad K, Klaaborg KE, Hjortdal V (2016). Prosthetic valve endocarditis after transcatheter aortic valve implantation-diagnostic and surgical considerations. J Thorac Dis.

[29] Takimoto S, Minakata K, Yamazaki K (2015). Successful surgical aortic valve replacement for prosthetic valve infective endocarditis following transcatheter aortic valve implantation. J Cardiol Cases.

[30] Wong DR, Boone RH, Thompson CR (2009). Mitral valve injury late after transcatheter aortic valve implantation. J Thorac Cardiovasc Surg.

[31] Comoglio C, Boffini M, El Qarra S (2009). Aortic valve replacement and mitral valve repair as treatment of complications after percutaneous CoreValve implantation. J Thorac Cardiovasc Surg.

[32] Sarı C, Durmaz T, Karaduman BD (2016). Prosthetic valve endocarditis 7 months after transcatheter aortic valve implantation diagnosed with 3D TEE. Hellenic J Cardiol.

[33] Castiglioni A, Pozzoli A, Maisano F, Alfieri O (2012). Endocarditis after transfemoral aortic valve implantation in a patient with Osler-Weber-Rendu syndrome. Interact CardioVasc Thorac Surg.

[34] Carrel T, Eberle B (2019). Candida endocarditis after TAVR. N Engl J Med.

[35] Morioka H, Tokuda Y, Oshima H (2019). Fungal endocarditis after transcatheter aortic valve replacement (TAVR): case report and review of literature. J Infect Chemother.

[36] Head SJ, Dewey TM, Mack MJ (2001). Fungal endocarditis after transfemoral aortic valve implantation. Catheter Cardiovasc Interv.

[37] Guenther SPW, Pichlmaier MA, Bagaev E (2016). Immediate, early and late failure after transcatheter aortic valve implantation: how to deal with the inoperable?. J Heart Valve Dis.

[38] Moufarrej R, Aljaberi N (2018). Prosthetic valve endocarditis secondary to Corynebacterium following transcatheter aortic valve implantation: a case report. Eur Heart J.

[39] Seeburger J, Weiss G, Borger MA, Mohr FW (2013). Structural valve deterioration of a CoreValve prosthesis 9 months after implantation. Eur Heart J.

[40] Zytowski M, Erb M, Albes JM, Hartrmpf M (2013). Infective endocarditis 4 months after transapical aortic valve implantation with Edwards Sapien XT. Eur J Cardiothorac Surg.

[41] Rodés-Cabau J, Webb JG, Cheung A (2012). Long-term outcomes after transcatheter aortic valve implantation: insights on prognostic factors and valve durability from the Canadian multicenter experience. J Am Coll Cardiol.

[42] Waksman R, Corso PJ, Torguson R (2019). Transcatheter aortic valve replacement in low-risk patients: one-year results from the LRT trial. JACC Cardiovasc Interv.

[43] Neragi-Miandoab S, Westbrook B, Flynn J, Blakely J, Baribeau Y (2015). Prosthetic valve endocarditis five months following transcatheter aortic valve implantation and review of literature. Heart Surg Forum.

[44] Dapás JI, Rivero C, Burgos P, Vila A (2016). Pseudomonas aeruginosa infective endocarditis following aortic valve implantation: a note of caution. Open Cardiovasc Med J.

[45] Latib A, Naim C, De Bonis M (2014). TAVR-associated prosthetic valve infective endocarditis: results of a large, multicenter registry. J Am Coll Cardiol.

[46] Chourdakis E, Koniari I, Hahalis G, Kounis NG, Hauptmann KE (2017). Early prosthetic valve endocarditis after transcatheter aortic valve implantation with periannular complication. J Geriatr Cardiol.

[47] Orban M, Sinnecker D, Mair H (2013). Transcatheter aortic-valve endocarditis confirmed by transesophageal echocardiography. Circulation.

[48] Spartera M, Schiavo LM, Sanvito F, Colombo A (2016). Early degeneration and endocarditis in a transcatheter heart valve. Eur Heart J.

[49] Raschpichler M, Seeburger J, Strasser RH, Misfeld M (2014). Corevalve prosthesis causes anterior mitral leaflet perforation resulting in severe mitral regurgitation and subsequent endocarditis. Eur Heart J.

[50] Ruchonnet EP, Roumy A, Rancati V, Kirsch M (2019). Prosthetic valve endocarditis after transcatheter aortic valve implantation complicated by paravalvular abscess and treated by pericardial patches and Sutureless valve replacement. Heart Surg Forum.

[51] Zhigalov K, Khokhlunov M, Szczechowicz M (2020). Right anterior minithoracotomy for endocarditis after transcatheter aortic valve replacement. Ann Thorac Surg.

[52] Kuwata S, Taramasso M, Maisano F, Weber A (2017). 22. Infective endocarditis after transcatheter aortic valve implantation with LOTUS valve. Eur Heart J.

[53] Bagozzi L, Bottio T, Gerosa G (2019). Rescue aortic root replacement for endocarditis after TAVR. Ann Thorac Surg.

[54] Vollenbroich R, Wenaweser P, Macht A (2019). Long-term outcomes with balloon-expandable and self-expandable prostheses in patients undergoing transfemoral transcatheter aortic valve implantation for severe aortic stenosis. Int J Cardiol.

[55] Barbanti M, Costa G, Zappulla P (2018). Incidence of long-term structural valve dysfunction and bioprosthetic valve failure after transcatheter aortic valve replacement. J Am Heart Assoc.

[56] Ando T, Ashraf S, Villablanca PA (2019). meta-analysis comparing the incidence of infective endocarditis following transcatheter aortic valve implantation versus surgical aortic valve replacement. Am J Cardiol.

[57] Mylotte D, Andalib A, Thériault-Lauzier P (2015). Transcatheter heart valve failure: a systematic review. Eur Heart J.

[58] Amat-Santos IJ, Ribeiro HB, Urena M (2015). Prosthetic valve endocarditis after transcatheter valve replacement: a systematic review. JACC Cardiovasc Interv.

[59] Moriyama N, Laakso T, Biancari F (2019). Prosthetic valve endocarditis after transcatheter or surgical aortic valve replacement with a bioprosthesis: results from the FinnValve Registry. EuroIntervention.

[60] Habib G, Erba PA, Iung B, EURO-ENDO Investigators (2019). Clinical presentation, aetiology and outcome of infective endocarditis. Results of the ESC-EORP EURO-ENDO (European infective endocarditis) registry: a prospective cohort study. Eur Heart J.

[61] Sugimura Y, Katahira S, Lopez Lopez R, Rellecke P, Lichtenberg A, Akhyari P (2019). Surgical aortic valve replacement due to infective endocarditis after transcatheter aortic valve implantation with the self-expanding Portico valve prosthesis. Ann Cardiothorac Surg.

[62] Gupta R, Ranchal P, Raza A, Sayed A, Pattarkine R, Dhand A (2019). Successful treatment of transcatheter aortic valve replacement infective endocarditis presenting with aortic root abscess in an Immunocompromised host. Am J Ther.

[63] Jawitz OK, Gulack BC, Grau-Sepulveda MV (2020). Reoperation after Transcatheter aortic valve replacement: an analysis of the society of thoracic surgeons database. JACC Cardiovasc Interv.

[64] Mihos CG, Capoulade R, Yucel E, Picard MH, Santana O (2017). Surgical versus medical therapy for prosthetic valve endocarditis: a meta-analysis of 32 studies. Ann Thorac Surg.

[65] Leontyev S, Davierwala PM, Krögh G (2016). Early and late outcomes of complex aortic root surgery in patients with aortic root abscesses. Eur J Cardiothorac Surg.

[66] Malvindi PG, Mikus E, Caprili L (2019). Aortic valve endocarditis complicated by proximal false aneurysm. Ann Cardiothorac Surg.

[67] Fukuhara S, Wu X, Deeb GM (2020). Surgical explant of Transcatheter aortic bioprosthesis: results and clinical implications from the Society of Thoracic Surgeons adult cardiac database analysis. J Am Coll Cardiol.

