# Supplementary material for: Surgical treatment of transcatheter aortic valve infective endocarditis
Source: Neth Heart J. 2020 Oct 6;29(2):71–7. doi: 10.1007/s12471-020-01494-y (PMC7843714; doi:10.1007/s12471-020-01494-y)
Supplement: Supplementary file 2 — PubMed research June 2020 [file 12471_2020_1494_MOESM2_ESM.docx]

**Electronic Supplementary Material**

PubMed research June 2020

- transcatheter[All Fields] AND ("aortic valve"[MeSH Terms] OR ("aortic"[All Fields] AND "valve"[All Fields]) OR "aortic valve"[All Fields]) AND ("endocarditis"[MeSH Terms] OR "endocarditis"[All Fields])
  - Results 306
- (tavi[All Fields] AND ("endocarditis"[MeSH Terms] OR "endocarditis"[All Fields])
  - Results 93
- (tavr[All Fields] AND ("endocarditis"[MeSH Terms] OR "endocarditis"[All Fields])
  - Results 70
- (TAVI[Title/Abstract] OR Transcatheter aortic valve[Title/Abstract]) AND ("infections"[MeSH Terms] OR "infections"[All Fields] OR "infection"[All Fields])
  - Results 187
- transcatheter[All Fields] AND ("aortic valve"[MeSH Terms] OR ("aortic"[All Fields] AND "valve"[All Fields]) OR "aortic valve"[All Fields]) AND ("infections"[MeSH Terms] OR "infections"[All Fields] OR "infection"[All Fields])
  - 228
- tavi[All Fields] AND ("infections"[MeSH Terms] OR "infections"[All Fields] OR "infection"[All Fields])
  - Results 96
- transcatheter[All Fields] AND ("aortic valve"[MeSH Terms] OR ("aortic"[All Fields] AND "valve"[All Fields]) OR "aortic valve"[All Fields]) AND explantation[All Fields]
  - Results 59
- tavi[All Fields] AND explantation[All Fields]
  - Results 12
- tavr[All Fields] AND explantation[All Fields]
  - Results 11
- transcatheter[All Fields] AND ("aortic valve"[MeSH Terms] OR ("aortic"[All Fields] AND "valve"[All Fields]) OR "aortic valve"[All Fields]) AND retrieval[All Fields]
  - Results 175
- tavi[All Fields] AND retrieval[All Fields]
  - Results 76
- tavr[All Fields] AND retrieval[All Fields]
  - Results 47
- transcatheter[All Fields] AND ("aortic valve"[MeSH Terms] OR ("aortic"[All Fields] AND "valve"[All Fields]) OR "aortic valve"[All Fields]) AND explant[All Fields]
  - Results 59
- tavi[All Fields] AND explant[All Fields]
  - Results 12
- tavr[All Fields] AND explant[All Fields]
  - Results 11
