# Supplementary material for: Surgical treatment of transcatheter aortic valve infective endocarditis
Source: Neth Heart J. 2020 Oct 6;29(2):71–7. doi: 10.1007/s12471-020-01494-y (PMC7843714; doi:10.1007/s12471-020-01494-y)
Supplement: Supplementary file 3 — Tab. 1 List of full-text papers included in the review [file 12471_2020_1494_MOESM3_ESM.docx]

**Electronic Supplementary Material**

**Tab. 1** List of full-text papers included in the review

| **Author, date** | **Number of patients** | **Age**  **(y)** | **Gender** | **Previous cardiac surgery** | **Type**  **of**  **valve** | **Cultures** | **Preoperative Details** | **Interval**  **time**  **(months)** | **Surgical operation**  **and findings** | **In-hospital outcomes** | **Follow-up** |
| --- | --- | --- | --- | --- | --- | --- | --- | --- | --- | --- | --- |
| Santarpino  2013  (25) | 1 | 83 | F | No | Sapien 23 | E. faecalis | EuroSCORE 25%  Acute kidney injury  Fever and sepsis after 1 week of antibiotic therapy  AV Vegetations and annular abscess | 6 | Emergency operation  SAVR Mitroflow 23 mm  + annular patch | Extubated in POD 5  ICU LOS 18 days  Discharged | - |
| Seok Koh  2014  (26) | 1 | 85 | M | No | Sapien 26 | S. angiosus | Presentation with fever and cerebral stroke  Recurrent cerebral stroke after 2 weeks of antibiotic therapy  AV Vegetations | 12 | SAVR bio + annular patch  No signs of endothelization | Discharged after 4 weeks with minor neurologic sequelae | - |
| Wilbring  2014  (27) | 1 | 76 | M | SAVR | Sapien 23 | Negative | Log ES 61%  Persistent fever  Aortic root abscess and TV vegetation | 48 | ARR bioconduit + annular reconstruction + TV repair    Salvage operation | Death after surgery due to persistent sepsis | - |
| Martínez-Sellés  2016  (20) | 1 | 79 | M | No | CoreValve | S. epidermidis | Presentation with heart failure  AV Vegetations and aortic root abscess | 4 | ARR | Discharged  Death within one month from operation due to cardiogenic shock and heart block | - |
| Ahmad K  2016 (28) | 1 | 80 | F | No | Sapien XT 23 | E. faecalis | Fever, abdominal pain and vaginal bleeding  After 6 weeks of antibiotics she presented with sepsis, neurologic deficit and spleen abscess  AV Vegetations | 6 | SAVR Perimount 23 mm | Discharged | - |
| Takimoto  2015 (29) | 1 | 80 | M | No | Sapien XT 26 | S. sanguis | Fever and asymptomatic cerebral embolism  Refractory endocarditis after 4 weeks of antibiotic treatment  AV and MV vegetations | 4 | SAVR Perimount 21 mm  + vegetectomy of MV leaflets | Discharged | - |
|  |  |  |  |  |  |  |  |  |  |  |  |
| **Author, date** | **Number of patients** | **Age**  **(y)** | **Gender** | **Previous cardiac surgery** | **Type**  **of**  **valve** | **Cultures** | **Preoperative Details** | **Interval**  **time**  **(months)** | **Surgical operation**  **and findings** | **In-hospital outcomes** | **Follow-up** |
| Wong  2009  (30) | 1 | 88 | M | CABG | Sapien 26 | S. angiosus | Fever  Moderate paravalvular AR  Rupture of AMVL | 11 | SAVR bio 25 mm + mitral valve repair  Incomplete endothelization | Course complicated by renal failure,  pneumonia, delirium, and dysphagia  Discharged | - |
| Comoglio  2009  (31) | 1 | 66 | M | No | CoreValve | Corynebacterium | Fever  Paravalvular AR, False aneurysm, AV vegetations,  Rupture of AMVL | 3 | SAVR bio 23 mm + mitral valve repair | Discharged | - |
| San  2016 (32) | 1 | 75 | F | No | Sapien XT 26 | E. faecium | Heart failure refractory to medical therapy  AV vegetation 11 mm | 7 | SAVR bio 21 mm | Death after surgery due to low cardiac output syndrome | - |
| Castiglioni  2012 (33) | 1 | 73 | M | No | Sapien XT 26 | Negative | Severe paravalvular leak, Aortic root abscess | 12 | SAVR Perimount 27 mm | Discharged | Alive at 6 months |
| Carrel  2019 (34) | 1 | 76 | M | No | - | Candida parapsilosis | Fever  Large obstructive AV vegetation | 9 | SAVR | Wound infection  Discharged | Died from pneumonia  at 3 months |
| Morioka  2019 (35) | 1 | 80 | M | No | Sapien 3 23 | Candida parapsilosis | Fever, Persistent candidemia  AV vegetation | 4 | SAVR Magna Ease | Discharged | Alive at 6 months |
| Head  2011  (36) | 1 | 78 | M | No | Sapien | Histoplasma capsulatum | STS score 18%  AV vegetation | 2 | SAVR  Signs of endothelization | Acute kidney injury  Discharged | Alive at 9 months |
| Guenther  2016  (37) | 7 | 81 | M | CABG | Sapien 4 patients  Sapien 3 2 patients  Corevalve 1patient | Enterococus faecalis 2 patients  Propionibacterium acnes 1 patient  S. salivarius 1 patient  Coagulase-negative  Staphylococcus 1 patient  St. epidermidis 1 patient  St. aureus 1 patient | ESII 43.04% | 52 | SAVR | Discharged | - |
|  |  | 70 | M | No |  |  | ESII 8.36% | 12 |  | Discharged |  |
|  |  | 71 | M | No |  |  | ESII 7.96% | 4 |  | Discharged |  |
|  |  | 77 | M | SAVR |  |  | ESII 12.23% | 4 |  | Discharged |  |
|  |  | 41 | M | SAVR |  |  | ESII 4.79 | 49 |  | Discharged |  |
|  |  | 83 | M | No |  |  | ESII 6.01% | 6 |  | Discharged |  |
|  |  | 79 | F | No |  |  | ESII 36.59 | 25 |  | Discharged |  |
| **Author,**  **date** | **Number of patients** | **Age**  **(y)** | **Gender** | **Previous cardiac surgery** | **Type**  **of**  **valve** | **Cultures** | **Preoperative Details** | **Interval**  **time**  **(months)** | **Surgical operation**  **and findings** | **In-hospital outcomes** | **Follow-up** |
| Moufarrej  2018  (38) | 1 | 87 | F | No | CoreValve | Corynebacterium amycolatum | Cerebral embolism, aspiration pneumonia  Vegetations and Aortic root pseudoaneurysm | 6 | Homograft ARR | Chylothorax,  Pneumonia  Septic shock  Discharged | Alive at 18 months |
| Seeburger  2013 (39) | 1 | 82 | M | SAVR | CoreValve 26 | - | Severe aortic valve stenosis | 9 | Freestyle 25 ARR | Discharged | - |
| Zytowski  2012 (40) | 1 | 84 | M | No | Sapien XT | Enterococcus durans | Vegetations, Annular abscess | 4 | - | - | - |
| Rodes-Cabau  2012 (41) | 2 | - | - | - | - | - | - | 7 | - | - |  |
|  |  |  |  |  |  |  |  | 13 |  |  |  |
| Waksman  2019 (42) | 2 | - | - | - | - | - | - | 3 | SAVR | Discharged |  |
|  |  |  |  |  |  |  |  | 7 | SAVR | Discharged |  |
| Amat-Santos  2015  (14) | 3 | 87 | M | Yes | Balloon expandable | S. angiosus | Mitral perforation  Log ES 35% | 11 | SAVR+MVr | Discharged | - |
|  |  | 70 | F | Yes | Balloon expandable | Negative | Cerebral stroke  Log ES 23% | 16 | SAVR | Death after surgery | - |
|  |  | 69 | M | No | Balloon expandable | S. epidermidis + enterococci | Log ES 38% | 2 | SAVR | Discharged | - |
| Neragi-Miandoab  2014  (43) | 1 | 65 | F | No | Sapien | Enterococcus faecalis | Upper GI bleeding  AV vegetations  Annular abscess | 4 | SAVR Mitroflow 21 | Respiratory failure  Sepsis  Death after surgery due to necrotizing pancreatitis | - |
| Dapas  2016  (44) | 1 | 62 | F | No | Jena Valve | P. aeruginosa | Peripheral embolism  Annular abscess  Log ES 78% | 2 | SAVR  Homograft | Discharged | Alive at 12 months |
| Regueiro  2016 (13) | 27 | - | - | - | - | - | - | - | - | Mortality 37% | - |
| Latib  2014 (45) | 3 | - | - | - | - | - | - | - | - | Mortality 67% | - |
| Salaun  2018  (21) | 2 | 51 | M | No | Sapien 23 | St. bovis | AV vegetations | 30 | SAVR bio | Discharged | Alive at 27 months |
|  |  | 80 | M | No | Sapien 3 29 | S. anginosus | Annular abscess | 8 | SAVR bio | Discharged | Alive at 45 months |
| Chourdakis  2017  (46) | 1 | 77 | F | No | Sapien XT | S. aureus | Septic arthritis  AV and MV vegetations | 1 | SAVR | Postoperative progression of endocarditis with aorto-LA fistula and MR | - |
| **Author,**  **date** | **Number of patients** | **Age**  **(y)** | **Gender** | **Previous cardiac surgery** | **Type**  **of**  **valve** | **Cultures** | **Preoperative Details** | **Interval**  **time**  **(months)** | **Surgical operation**  **and findings** | **In-hospital outcomes** | **Follow-up** |
| Olsen  2015  (15) | 1 | 76 | M | No | CoreValve | S. salivarius | AV and MV vegetations  MV perforation | 30 | SAVR bio + MVr + TV annuloplasty | Discharged | - |
| Orban  2013  (47) | 1 | 70 | M | No | CoreValve | S. epidermidis | Peripheral embolism  AV vegetations  Annular abscess | 12 | SAVR  Hancock II 25 | Discharged after 2 weeks | - |
| Spartera  2016 (48) | 1 | 83 | F | No | Sapien 3 23 | S. gallolyticus | Fever, Heart failure  AV vegetations | 12 | SAVR Carpentier-Edwards 21 | Discharged | - |
| Raschpichler  2013 (49) | 1 | 84 | M | No | CoreValve | S. epidermidis | Fever, Heart failure  AV and MV vegetations  MV perforation | 6 | SAVR Trifecta 27 +  MVR Epic 33 | Discharged | - |
| Ruchonnet  2019 (50) | 1 | 84 | F | No | Symetis | S. aureus | Annular abscess and right atrium fistula | 1 | SAVR Perceval S + annular repair | Discharged | Alive at 12 months |
| Mangner  2018 (23) | 20 | 77 | M 13 patients  F 7 patients | SAVR 1 patient | Self-expandable 7 patients  Balloon expandable 13 patients | Coagulase-positive  Staphylococcus  30%  Coagulase-negative  Staphylococcus  10%  Enterococcus 40%  Streptococcus 15%  Fungi 5% | Annular abscess 35% | 7.7 (2–19) | Isolated SAVR 30%  MVR 35%  ARR 10% | Mortality 50% | Mortality 1-y 65% |
| Zhigalov  2020 (51) | 1 | 75 | M | No | Sapien 29 | Streptococcus sanguis | Refractory heart failure and sepsis  Vegetation  Aortic regurgitation | 2 | SAVR Perceval 27 | Discharged | - |
| Kuwata  2016 (52) | 1 | 84 | M | No | Lotus 25 | Streptococcus gordonii | Fever  Vegetation  Peripheral embolism | 12 | SAVR Carpentier-Edwards 23 | Discharged | - |
| Bagozzi  2020 (53) | 1 | 58 | M | No | CoreValve | Staphylococcus lugdunensis | Annular abscess | - | ARR Biointegral 27 | Discharged | Alive at 12 months |
| Sugimura 2020 (61) | 1 | 83 | M | No | Portico 27 | S. aureus | Heart failure  Severe paravalvular leak | 4 | SAVR Magna Ease 23 + Non-coronary sinus replacement  Partial endothelization | Discharged | - |
| **Author,**  **date** | **Number of patients** | **Age**  **(y)** | **Gender** | **Previous cardiac surgery** | **Type**  **of**  **valve** | **Cultures** | **Preoperative Details** | **Interval**  **time**  **(months)** | **Surgical operation**  **and findings** | **In-hospital outcomes** | **Follow-up** |
| Gupta 2020  (62) | 1 | 58 | M | No | - | St. agalactiae | Fever  Annular abscess | 12 | ARR | Discharged | Alive at 12 months |
| Jawitz 2020  (63) | 12 | - | - | - | - | - | - | - | SAVR | Mortality 25% | - |

ARR: aortic root replacement; AMVL: anterior mitral valve leaflet; AV: aortic valve; ES: EuroSCORE; MV: mitral valve; MVr: mitral valve repair; MVR: mitral valve replacement; SAVR: surgical aortic valve replacement; ViV: valve-in-valve
